# Karyotype Plasticity in Crickets: Numerical, Morphological, and Nucleolar Organizer Region Distribution Pattern of *Anurogryllus* sp.

**DOI:** 10.1673/031.010.8701

**Published:** 2010-07-02

**Authors:** Marielle Cristina Schneider, Adilson Ariza Zacaro, Amilton Ferreira, Doralice Maria Cella

**Affiliations:** ^1^ Universidade Federal de São Paulo, UNIFESP, Departamento de Ciências Biológicas, Diadema, Säo Paulo, Brazil; ^2^ Universidade Federal de Viçosa, UFV, Departamento de Biologia Geral, Viçosa, Minas Gerais, Brazil; ^3^ Universidade Estadual Paulista, UNESP, Instituto de Biociências, Departamento de Biologia, Rio Claro, São Paulo, Brazil

**Keywords:** centric fusion, cytogenetic, meiosis, secondary constriction, translocation

## Abstract

Within the Orthopteran species, those of the suborder Ensifera have been rarely studied from the cytogenetic point of view, mainly due to the difficulties for taxonomic identification of its species. The Gryllidae is the second largest family of this suborder and possesses some genera, such as *Anurogryllus*, that occur only on the American continents. The aim of this work was to determine the karyotype characteristics, the meiotic chromosome behaviour, and the nucleolar organizer region (NOR) pattern of *Anurogryllus* sp (Orthoptera: Gryllidae). In the analyzed sample, high levels of numerical, morphological, and NORs polymorphisms were detected. Within five distinct karyotypes that were found, the basic karyotype of *Anurogryllus* sp. showed 2n(♂) = 22 + X0 with acrocentric autosomes and a metacentric X sex chromosome; furthermore, a conspicuous secondary constriction related to the NOR was present along the entire short arm on pair 5. The other four types of karyotypes arose from centric fusions between elements of pairs 1/3, 2/6, 4/7 and a NOR partial translocation from pair 5 onto the long arm terminal region of one element of the fused pair 2/6. Such intraspecific variability and the consequences of high levels of polymorphism are discussed, leading to conjectures about the mechanisms that led to these chromosome rearrangements.

## Introduction

The order Orthoptera possesses more than 20,000 worldwide species, but its highest diversity is concentrated in the tropical region (Gwynne et al. 1996; Eades and Otte 2006). This order is divided into two suborders: Caelifera, with approximately 11,000 species, including the grasshoppers and the locusts, and Ensifera, which has around 9,000 species, including the crickets and the katydids (Gwynne et al. 1996). Though these two suborders have an almost equal number of described species, caeliferans have been more extensively studied from the cytogenetic viewpoint (Hewitt 1979; Bhunya and Das 1985; Das and Das 1991, 1992). This difference may be due, in part, to the lack of taxonomic identification of the ensiferan species. Because data regarding the external morphology and genitalia are not sufficient for description of new species, the use of other characteristics, such as behavioural and acoustical information, became necessary (Alexander 1962a, 1962b; Stumpner and Meyer 2001). Additionally, the phylogenetic relationships between the families and subfamilies of Ensifera are still poorly understood and there is no consensus on phylogenetic hypotheses previously proposed for the relationships between the families (Jost and Shaw 2006).

Taking into account the current classification proposed by Eades and Otte (2006), the suborder Ensifera is composed of 12 living families (Anostatomatidae, Cooloolidae, Grylacrididae, Gryllidae, Gryllotalpidae, Mogoplistidae, Myrmecophilidae, Prophalangopsidae, Rhaphidophoridae, Schizodactylidae, Stenopelmatidae, and Tettigonidae). Of these families, Gryllidae is the second largest in number of representatives, possessing more than 3500 valid species. In this family, some genera have species distributed among almost all the continents, e.g. *Gryllus* Linnaeus 1758 and *Nemobius* Serville 1838, while others show a more restricted distribution, such as *Anurogryllus* Saussure 1877, which includes at least 11 species that were already taxonomically described and that occur only on the American continents (Eades and Otte 2006). Certainly, this last genus has a high number of species to be identified, primarily from Brazilian fauna. The genus *Anurogryllus* is also interesting because of its sub-social behaviour in which females exhibit care for the offspring (Liebermann 1955; West and Alexander 1963).

In Gryllidae, the cytogenetic information showed that the diploid number ranged from 2n = 7 to 29, with 2n = 21 being most frequently reported for this taxon. The X0 sex chromosome system is the predominant type in this group; nevertheless, other simple or multiple systems, such as XY and X_1_X_2_Y, were also reported. As with the majority of orthopterans, the Gryllidae autosomes commonly exhibit the acrocentric morphology; unlike other orthopteran families, the X sex chromosome is metacentric (Ashwath and Aswathanarayana 1979; Hewitt 1979; Warchalowska-Sliwa 1980; Baccetti and White 1983; Bhunya and Das 1985; Lamborot and Alvarez-Sarret 1985; Davenport 1986; Das 1989; Das and Das 1991, 1992; Warchalowska-Sliwa et al. 1997; Zefa 1999; Mesa and Zefa 2004; Ferreira and Cella 2006).

Considering the lack of cytogenetic studies in crickets from Brazilian fauna using techniques of standard and differential staining, the aim of this work was to determine the karyotype characteristics, the chromosomal behaviour during meiosis, and the nucleolar organizer region (NOR) distribution pattern in *Anurogryllus* sp. (Orthoptera: Gryllidae). The obtained data were compared with those of related species to establish the basic karyotype features of *Anurogryllus* sp. and to explain the remarkable variability of chromosome number, morphology, and NOR distribution pattern found in the investigated sample.

## Materials and Methods

Three adult crickets (one male and two females) were collected in an area of vegetation of the Universidade Estadual Paulista, UNESP, Rio Claro (22° 24′ S, 47° 33′ W), State of São Paulo, Brazil. These specimens showed very similar morphological characteristics and were identified by Alejo Mesa as *Anurogryllus* sp. One female was placed in a plastic container identified as A, and the two others, a female and male, were kept in a separate plastic box, identified as B. These containers had a soil layer, food (oats and spring greens), and water, and they were kept at a temperature of approximately 25° C. Female A was already fertilized when it was caught in the field, and Female B mated in the laboratory. Both females laid eggs in soil galleries. Not all eggs were useful for cytological observation because some of them did not develop embryos. Five eggs from Female A (Offspring A: three males and two females) and six eggs from Female B (Offspring B: six females) were used for making embryonic chromosome preparations. Moreover, some of the Offspring A eggs were kept in the plastic container until the specimens (three males) reached the last nymphal instar; all living male nymphs were dissected, and the testes were used to obtain the mitotic and meiotic chromosome preparations. Attempts were made to obtain cell division from testes of adult specimens. However, the observed lack of mitotic and meiotic cells, and the high quantity of spermatozoa in the gonadal preparations may indicate that spermatogenesis in this species probably occurs in the pre-adult phase or that the male adult specimens were too old for cytogenetic studies. The analysis of chromosomes of pre-adult specimens collected from the field was not possible due to the difficulties in taxonomic identification of the individuals in this instar. The midgut caecum of adult specimens was also alternatively used to make mitotic chromosome preparations; nevertheless, only the Female B caecum provided some results.

The cytological preparations (embryos, testes, and midgut caecum) were processed according to Webb et al. (1978) and standard stained with Giemsa solution (3% of commercial Giemsa and 3% of phosphate buffer pH 6.8 in distilled water). After analysis of the chromosomal preparations standard stained (Giemsa), some were also submitted to the silver nitrate impregnation technique (Howell and Black 1980) to detect the nucleolar organizer regions (AgNOR). The chromosome morphology was determined according to nomenclature proposed by Levan et al. (1964).

## Results

### Standard staining

**Figure 1.**
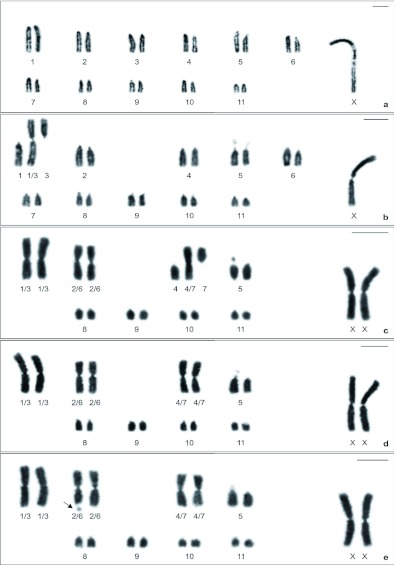
Karyotypic diversity of *Anurogryllus* sp. detected in mitotic cells stained with Giemsa. A: Karyotype 1, 2n (♂) = 22 + X0. B: Karyotype II, 2n (♂) = 21 + X0, showing the heterozygous state for the centric fusion 1/3. C: Karyotype III, 2n (♀) = 17 + XX, demonstrating the homozygous form for the centric fusion 1/3 and 2/6, and the heterozygous condition for the centric fusion 4/7. D–E: Karyotype IV and V, respectively, 2n (♀) = 16 + XX, revealing the homozygous condition for the centric fusion 1/3, 2/6, and 4/7. In E, observe the heteromorphic secondary constriction on the long arm distal region of the 2nd metacentric pair (arrow). In all karyotypes, the pair 5 showed a secondary constriction on the whole short arm. Scale bar = 5 µm. High quality figures are available online.

The analysis of mitotic cells of embryos, male nymphs, and female adult of *Anurogryllus* sp. revealed intraspecific karyotypical diversity due to numerical and structural variations of autosomes (Figure 1). However, a predominance of autosomes with acrocentric morphology, an invariable number of autosomal arms equal to 22, and a single sex determination system of the X0/XX type were detected in all specimens. The X chromosome was always easily identified by virtue of its large size and metacentric morphology. Furthermore, variation in the length of the short arms of the 5th pair chromosomes was observed in the great majority of the cells of all specimens; but the analysis of approximately 60 mitotic metaphases revealed that this heteromorphism was apparent and that it occurred due to the presence of a secondary constriction.

Considering all chromosomal variations encountered in the sample investigated, five distinct karyotypes could be recognized, which were described as follows:

**Karyotype I.** This karyotype was found in four embryos (two males and two females) of Offspring A and was characterized by diploid number 2n = 22 + X0 in males and 2n = 22 + XX in females. In this karyotype, all autosomic pairs were formed by acrocentric chromosomes that gradually decrease in size (Figure 1A).

**Karyotype II.** Karyotype 2n = 21 + X0 was encountered in only one male embryo of Offspring A and was composed of nine homomorphic acrocentric autosome pairs (pair 2 and pairs 4–11) and three heteromorphic chromosomes, that is, one metacentric of large size and two acrocentric of different sizes. These three unpaired chromosomes corresponded to a heteromorphic state for centric fusion that involved elements of both pairs 1 and 3 (Figure 1B).

**Karyotype III.** Only one female embryo of Offspring B showed a karyotype 2n = 17 + XX constituted of autosomal chromosomes with the following morphology: five acrocentric pairs (pair 5 and pairs 8–11), two metacentric pairs of large size, and three heteromorphic chromosomes (one metacentric of large size and two acrocentric of distinct sizes). The first and the second metacentric pairs represent the homomorphic state for a centric fusion between acrocentric chromosomes of pairs 1 and 3 and pairs 2 and 6, respectively. This karyotype also showed three heteromorphic chromosomes, indicating the heteromorphic condition for a centric fusion between chromosomes of the 4th and 7th pairs (Figure 1C).

**Karyotype IV.** Karyotype 2n = 16 + X0 in males and 2n = 16 + XX in females was observed in most of the samples investigated, occurring in three male nymphs and in three female embryos of the Offspring A and B, respectively. The autosomal set comprised five acrocentric pairs (pair 5 and pairs 8–11) and three large metacentric pairs that exhibited a slight difference in length. These three large metacentric pairs corresponded to a homomorphic state for a centric fusion between acrocentric chromosomes of pairs 1 and 3, pairs 2 and 6, and pairs 4 and 7 (Figure 1D).

**Karyotype V.** This karyotype was encountered in two female embryos of the Offspring B and showed very similar characteristics to those above-mentioned for karyotype IV, only differing by the occurrence of a distal secondary constriction on the long arm of the 2nd pair (homomorphic form for the centric fusion between chromosomes of pairs 2 and 6). It is worth pointing out that the secondary constriction was always verified in a heteromorphic state (Figure 1E).

The metaphases of the Female B midgut caecum preparations also denoted the occurrence of 2n = 16 + XX. The karyotype of this female could not be precisely determined as IV or V as the chromosomes showed high degree of condensation, making impossible the detection of the secondary constriction on 2nd pair.

**Figure 2.**
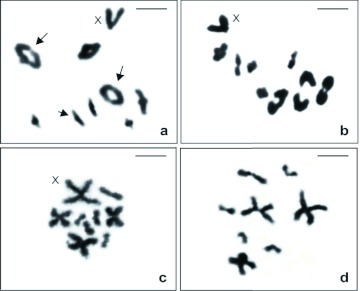
Meiotic cells of *Anurogryllus* sp. standard stained. A–B: Early and late metaphase I, respectively, 2n = 8II + X0. In A, note the autosomal bivalents with one (small arrow) or two (large arrow) terminal chiasmata. C–D: Metaphase II, with n = 8 + X and n = 8, respectively. Scale bar = 5 µm. High quality figures are available online.

The analysis of meiotic spermatocytes was accomplished in three male nymphs of Offspring A (Figure 2). In all these three specimens, the late prophase I and metaphase I cells revealed the meioformula 8II+X0, confirming the diploid number and the type of sex determination system verified in mitotic metaphases of individuals with karyotype IV. In diplotene and diakinesis cells, the three largest autosomal bivalents, certainly metacentrics, exhibited the ring shape, whereas the five others showed the rod shape; the X sex chromosome was the most easily recognized element because of its “V” shape and large size. The ring and rod shape of the autosomal bivalents during meiosis indicated, respectively, the occurrence of two and one terminal chiasmata. Metaphase II demonstrated two different haploid sets, n = 8 + X and n = 8, confirming the regular segregation of all chromosomes during the first meiotic division.

### Silver nitrate impregnation

**Figure 3.**
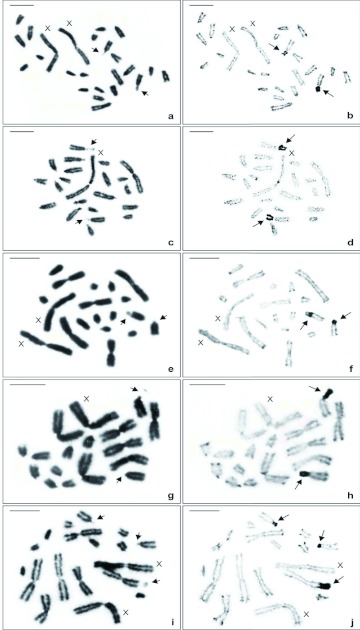
NOR pattern in mitotic cells of *Anurogryllus* sp. submitted to both standard staining (A, C, E, G, I) and silver nitrate impregnation (B, D, F, H, J). A–B: Female, 2n = 22 + XX. C–D: Male, 2n = 21 + X0. E–F: Female, 2n = 17 + XX. G–H: Male, 2n = 16 + X0. I–J: Female, 2n = 16 + XX. The small arrow indicates secondary constriction and the large arrow shows NOR. Scale bar = 5 µm. High quality figures are available online.

Mitotic cells with all types of numerical and structural chromosome variation, when submitted to silver impregnation, allowed the establishment of the distribution pattern of active NORs in *Anurogryllus* sp. In all embryos and nymphs, two conspicuous NORs were observed on the entire pair 5 short arms, being coincident with the secondary constrictions (Figure 3 A to J). In almost all analyzed cells of the specimens with karyotype V, an additional NOR coincident with the secondary constriction was detected on the long arm terminal region of the 2nd metacentric pair (Figures 3 I and J). This NOR was heteromorphic, as the secondary constriction observed in this chromosome, occurred on only one element of the pair.

## Discussion

Cytogenetic studies in Orthoptera have revealed a high diversity of examples of intra and interspecific karyotype variation. In general, these variations are result of chromosome rearrangements, such as translocations and centric fissions or fusions, which have mainly involved the autosomes and occasionally the sex chromosomes. Moreover, supernumerary chromosomes and extra chromosomal segments have also been registered in orthopteran species (Hewitt 1979; Henriques-Gil et al. 1984; López-Fernández et al. 1984; Bhunya and Das 1985; Lamborot and Alvarez-Sarret 1985; Davenport 1986; Colombo 1987; Das 1989; Fossey et al. 1989; Das and Das 1991; Mirol and Bidau 1991; Warchalowska-Sliwa et al. 1992; Eugênio and Cella 1996; Zefa 1999; Colombo et al. 2004). The mechanisms responsible for the chromosomal changes observed in orthopteran could have particular effects on karyotype evolution, taking into account that these could influence the frequency of recombination, fertility, and mating success (Hewitt 1979; López-Fernández et al. 1984; Colombo 1987, 1993a, 1993b; Fossey et al. 1989; Colombo et al. 2001, 2004).

In *Anurogryllus* sp., a high level of chromosome polymorphism was observed, allowing the determination of five distinct karyotypes. Considering that karyotype 2n = 22 + X0 seems to be the basic type for the family Gryllotalpidae (Hewitt 1979), which was recently determined to be a sister group to Gryllidae according to molecular data (Jost and Shaw 2006), and considering that autosomes with acro/telocentric morphology are the most frequently detected in species of Gryllidae as well as in the majority of the representatives of the suborder Ensifera (Ashwath and Aswathanarayana 1979; Hewitt 1979; Warchalowska-Sliwa 1980; Baccetti and White 1983; Bhunya and Das 1985; Lambort and Alvarez-Sarret 1985; Davenport 1986; Das 1989; Das and Das 1991, 1992; Warchalowska-Sliwa et al. 1997; Zefa 1999; Mesa and Zefa 2004; Ferreira and Cella 2006), it seems reasonable to accept that karyotype I (2n(♂) = 22+X0 with all autosomes acrocentric) is the pattern type for *Anurogryllus* sp. The karyotypes with lower diploid number and some metacentric autosomes probably originated by centric fusions and should be derived from the higher diploid number.

A comparative analysis made among the five distinct karyotypes indicated that metacentric chromosomes observed in karyotypes II, III, IV, and V probably evolved through centric fusions involving chromosomes of the pairs 1, 2, 3, 4, 6, and 7 of the less differentiated karyotype. Additionally, the fact that the sex chromosome system was always of the X0/XX type in all the karyotypes described here and that the X chromosome was unchanged in relation to its size and morphology, also reinforces the proposition that in the sample studied the chromosomal polymorphism only occurred by rearrangements that involved autosomes. In an unpublished M.Sc. dissertation, García (2000) also reported the occurrence of autosomal polymorphism in adult and nymph *Anurogrullus* sp. from the same population of the sample studied in this work. Although this author had not specified which chromosomes were rearranged, certain presented karyotypes seemed to be similar to some reported in this study.

According to Slijepcevic (1998), in the chromosomes of many mammalian species, there are at least three mechanisms already established that can lead to centric fusions: loss of the telomeric sequence by telomere shortening, loss of the telomeric sequence due to chromosome breakage, and telomere shortening caused by telomerase inactivation. In *Anurogryllus* sp. the use of advanced cytogenetic techniques, such as in situ hybridization with telomere probe, could supply data for understanding the molecular mechanisms responsible for centric fusions.

In species of the genus *Anurogryllus*, there is not much information about sexual behavior. However, West and Alexander (1963) suggested single mating per female in *Anurogryllus muticus* (De Geer 1773). If the hypothesis of single mating is considered for *Anurogryllus* sp., the Progenitors A should have the centric fusions 1/3, 2/6, and 4/7 in heterozygous state once some analyzed individuals of Offspring A revealed homomorphic karyotype for absence (karyotype I) or presence (karyotype IV) of centric fusions that involved pairs 1, 2, 3, 4, 6, and 7. In relation to the Progenitors B, the analysis of midgut caecum chromosome preparations showed that the female possessed a karyotype with the homomorphic condition for the centric fusions 1/3, 2/6, and 4/7. The male probably has the centric fusion 4/7 in the heterozygous state as their offspring showed homomorphic or heteromorphic karyotype for this fusion. Nevertheless, the obtained results were not enough to establish the general male B karyotype constitution.

The fact that all studied individuals of the Offspring A and B showed balanced karyotypes seems to point out the occurrence of some mechanisms that ensure the chromosome balanced segregation during meiosis and, consequently, the maintenance of the chromosomal rearrangements in the population. Furthermore, not only the homozygous offsprings seem to be viable, but also those heterozygous, considering that some progenitors could be heterozygous for the centric fusions. However, there is the possibility that the eggs with embryos having unbalanced karyotypes, whose development could fail, would become trophic eggs, increasing the chances of success of the offspring with balanced chromosomal polymorphism in population. The occurrence of trophic eggs was already reported in *A. muticus* (West and Alexander 1963).

A less condensed chromatin region, usually heteromorphic and similar to that detected in pair 5 of *Anurogryllus* sp., was also encountered in one autosomal pair of other species of crickets (Warchalowska-Sliwa 1980; Lamborot and Alvarez-Sarret 1985; Das and Das 1991; Zefa 1999; Mesa and Zefa 2004; Yoshimura 2005; Portugal and Mesa 2007). In *Anurogryllus* sp., the uncondensed chromatin region was representative of the NOR, considering that in all the studied specimens the short arm of both chromosomes of pair 5 was silver impregnated. However, in the majority of the cricket species there is no information about the correspondence of this less condensed chromosome region and NOR, with the exception of *Gryllus rubens* and *Gryllus* sp. that were detected by Yoshimura (2005) as NORs.

The observed differences in the size of the secondary constriction between the homologous chromosomes of *Anurogryllus* sp. can be explained in three ways: a) localization of the secondary constriction on the terminal region of the chromosome arm, which in virtue of its degree of condensation was not always detected in chromosomal preparations stained with Giemsa; b) differences in the transcriptional activity of the NORs; c) variability in the number of the ribosomal cistrons. However, the results obtained in this work revealed that in the cells stained with Giemsa, in which pair 5 seemed to be heteromorphic in relation to the size of the constriction, the NOR detected by silver impregnation possessed similar size in both chromosomes. Moreover, Zurita et al. (1998) verified, through a sequential procedure of silver impregnation and in situ hybridization (ISH) with an rDNA probe, that in the mammal *Talpa occidentalis* there was a relationship between the level of transcriptional activity of the NOR and the number of rDNA copies. This association between the AgNOR size and the signal detected by the FISH technique was also registered in some other groups, such as Pisces (Collares-Pereira and Ráb 1999) and Molusca (Cross et al. 2003). Thus, the heteromorphic condition of the pair 5 less condensed region detected in *Anurogryllus* sp. should not be due to the difference in the number of ribosomal cistrons.

The NOR number variation observed in the specimens with karyotype V due to an extra AgNOR coincident with the secondary constriction on the 2nd metacentric pair indicates that the karyotypical plasticity of *Anurogryllus* sp. is not only related to the rearrangements that involve entire chromosomes, but also translocation of specific chromosomal regions, that is, ribosomal cistrons. The variability in the number and localization of the NORs can occur due to partial or total translocations of the rDNA genes. According to some researchers, transposition has also been the cause of the NOR changes. Transposable elements were already encountered in association with the rDNA genes of many species, including *Apis mellifera, Bombyx mori, Drosophila melanogaster,* and the green pufferfish, *Tetraodon fluviatilis* (Jakubczak et al. 1990; Bigot et al. 1992; Mandrioli 2000). Furthermore, taking into account that ribosomes are needed in high quantity for cellular metabolism, the presence in the genome of multiple copies of ribosomal cistrons distributed in tandem or in different sites of the chromosomes could be an evolutionary advantage (Long and Dawid 1980).

In conclusion, all karyotype variations encountered in *Anurogryllus* sp. could indicate that this species supports several chromosomal rearrangements and that the genetic variability could be correlated not only with chiasmata and chromosome random segregation, but also by the presence of chromosome polymorphism of the centric fusion and/or translocation types. The remarkable karyotypic variability of *Anurogryllus* sp. could also indicate the occurrence of more than one species morphologically identical in the collection area, considering that diversified but sympatric karyotypes were found between the offspring of the two females analyzed. However, some karyotypes showed similar combinations of chromosome number and morphology between the Offspring A and B. Thus, the investigation of a high number of specimens belonging to distinct populations and the employment of techniques to highlight specific chromosomal regions should be done, with the aim of clarifying the chromosomal rearrangements that have occurred in this species and to verify if intrapopulational differences in the karyotypic characteristics of *Anurogryllus* sp. could be related to a species complex.

## Abbreviation

NOR: nucleolar organizer region
